# Detailed Analysis of Prebiotic Fructo- and Galacto-Oligosaccharides in the Human Small Intestine

**DOI:** 10.1021/acs.jafc.4c03881

**Published:** 2024-09-16

**Authors:** Mara P. H. van Trijp, Melany Rios-Morales, Madelon J. Logtenberg, Shohreh Keshtkar, Lydia A. Afman, Ben Witteman, Barbara Bakker, Dirk-Jan Reijngoud, Henk Schols, Guido J. E. J. Hooiveld

**Affiliations:** † Division of Human Nutrition and Health, Wageningen University, Wageningen 6708 WE, The Netherlands; ‡ Laboratory of Pediatrics, Center for Liver, Digestive and Metabolic Diseases, University of Groningen, University Medical Center Groningen, Groningen 9713 GZ, The Netherlands; § Laboratory of Food Chemistry, Wageningen University, Wageningen 6708 WG, The Netherlands; ∥ Department of Gastroenterology and Hepatology, Hospital Gelderse Vallei, Gelderland 6716 RP Ede, The Netherlands

**Keywords:** digestion, oligosaccharides, small intestine, ileum, prebiotics, lactose, human

## Abstract

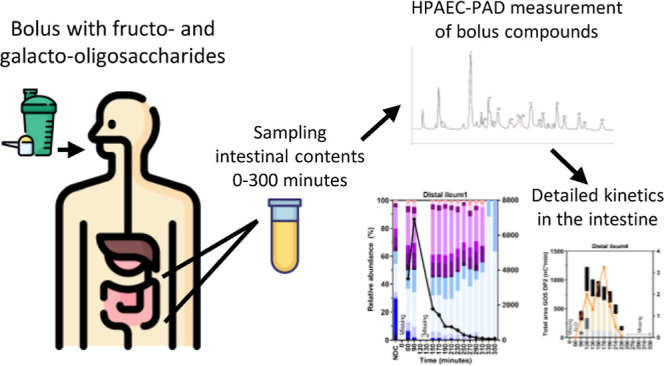

Galacto-oligosaccharides (GOS) and fructo-oligosaccharides (FOS) are food ingredients that improve human health, but their degradation throughout the human small intestine is not well understood. We studied the breakdown kinetics of FOS and GOS in the intestines of seven healthy Dutch adults. Subjects were equipped with a catheter in the distal ileum or proximal colon and consumed 5 g of chicory-derived FOS (degree of polymerization (DP) DP2–10), and 5 g of GOS (DP2–6). Postprandially, intestinal content was frequently collected until 350 min and analyzed for mono-, di-, and oligosaccharides. FOS and GOS had recoveries of 96 ± 25% and 76 ± 28%, respectively. FOS DP ≥ 2 and GOS DP ≥ 3 abundances in the distal small intestine or proximal colon matched the consumed doses, while GOS dimers (DP2) had lower recoveries, namely 22.8 ± 11.1% for β-D-gal-(1↔1)-α-D-glc+β-D-gal-(1↔1)-β-D-glc, 19.3 ± 19.1% for β-D-gal-(1 → 2)-D-glc+β-D-gal-(1 → 3)-D-glc, 43.7 ± 24.6% for β-D-gal-(1 → 6)-D-gal, and 68.0 ± 38.5% for β-D-gal-(1 → 4)-D-gal. Lactose was still present in the distal small intestine of all of the participants. To conclude, FOS DP ≥ 2 and GOS DP ≥ 3 were not degraded in the small intestine of healthy adults, while most prebiotic GOS DP2 was hydrolyzed in a structure-dependent manner. We provide evidence on the resistances of GOS with specific β-linkages in the human intestine, supporting the development of GOS prebiotics that resist small intestine digestion.

## 1 Introduction

Nondigestible carbohydrates (NDCs) are valuable food ingredients applied for their health benefits.^1^ Galacto-oligosaccharides (GOS) and fructo-oligosaccharides (FOS) are examples of soluble NDCs that serve as fermentable substrates for gut microbiota. Their degree of polymerization (DP) fractions ≥3 are also classified as dietary fibers.^2,3^ Moreover, GOS and FOS are prebiotics, which are defined as being ‘a substrate that is selectively utilized by the host microorganisms conferring a health benefit’.^4^ Both FOS and GOS are naturally present in various foods. However, they are also industrially produced as ingredients to add to foods or supplements to improve the nutritional value of the food and/or for human health purposes.^2^ For instance, FOS and GOS are added to infant formula to mimic the health effects of endogenous oligosaccharides in human milk,^5^ or added to foods to increase the fiber content for adults.^6^

Fructans, including inulin and oligofructose, are naturally found in foods such as whole grains, vegetables (e.g., garlic, artichoke), and fruits (e.g., bananas).^7−9^ FOS (DP 2–10) is produced via partial enzymatic hydrolysis of inulin that is extracted mainly from chicory roots.^10^ Alternatively, FOS (DP2–5) may be prepared from sucrose or fructose.^8^ FOS consist of a linear series of β-(2,1) linked fructose units, attached to a terminal fructose by a β-(2,1) bond (Fn series), or to a terminal alpha-d-glucose by an α-(2,1) bond (GFn series) at the nonreducing end, with a DP up to 10.^8,11^ Inulinases degrade FOS and can be classified into endo- and exoinulinases. Endoinulinases (2,1-β-D-fructan fructanohydrolase) split internal β-(2,1) fructofuranosyl linkages, whereas exoinulinases (β-d-fructohydrolase) split off fructose units at the terminal nonreducing end.^12^ Several microorganisms residing in the human gut possess these enzymes,^12^ whereas host enzyme sucrase-isomaltase in the small intestine can split sucrose (α-D-glc-(1 → 2)-β-D-fru)^13^ but not β-(2,1) linked fructose units.

GOS is naturally present in human milk,^14^ as well as in the generative part of plants such as beans or legumes (e.g., lentils, chickpeas).^15^ They can also be produced via hydrolysis and transgalactosylation of lactose by β-galactosidases.^16^ The production results in a mixture of galactose chains varying in DP (2–8), and linkages,^16^ namely β-(1,2), β-(1,3), β-(1,4), or β-(1,6), attached to a terminal galactose or glucose unit,^16−18^ or isomers with a (1↔1) linkage.^17^ The effects of GOS on the microbiota composition, intestinal immunity, and intestinal barrier function are dependent on monomer composition, DP, or linkage type.^19−21^ This highlights the importance of studying structure-dependent health effects. Degradation of GOS in the intestinal tract requires glycoside hydrolases, specifically β-galactosidases.^22,23^ Specific gut microorganisms contain β-galactosidases with different activities.^24^ One type of β-galactosidase, lactase, hydrolyzes lactose into galactose and glucose. Lactase is one of the only two β-galactosidases, next to the lysosomal enzyme β-galactosidase-1, that is also encoded by humans, and is attached to the intestinal brush border membrane.^25,26^ Its levels are decreased in early childhood and further decline during aging. The decline, however, varies among ethnic backgrounds, as for instance Northern European adults have persistent lactase activity.^27^

Despite the interest in FOS and GOS due to their potential health benefits, knowledge of their degradation in the human small intestine is limited. Developing and applying NDCs in foods is of interest because they have low caloric value, give a low or extended glycemic response, and can function as substrates for the colonic microbiota. There is a generally accepted view that NDCs pass through the small intestine without substantial modifications.^28^ Yet, some animal studies hint toward the start of NDC fermentation in the small intestine,^29,30^ and in vitro FOS and GOS can be fermented by ileostomy bacteria.^31^ Breakdown of FOS by human intestinal bacteria in vitro was shown to occur in a size-dependent manner.^31^ Moreover, FOS^32,33^ and 4′-galactosyllactose^34^ are resistant to digestion by the rat digestive enzymes of the GI-tract in vitro,^32−34^ but GOS with specific linkages was slightly digested in vitro by rat^33,35^ or pig digestive enzymes.^36^ Several studies investigated the resistance of FOS^37^ or inulin^38,39^ to degradation and absorption in the human small intestine. Chicory inulin and oligofructose were recovered in the ileostomy effluent of patients, suggesting minor losses due to hydrolysis or bacterial degradation during small intestinal passage.^39^ In another study in ileostomy patients, artichoke inulin was not fully recovered from the small intestine.^38^ Similarly, using intestinal aspiration in healthy volunteers, a minor fraction of chicory FOS was not recovered from the small intestine.^37^ However, a detailed analysis of the fate of individual FOS DP fractions was not provided. So far, no clinical trials studying GOS degradation in humans have been conducted. Consequently, there is a need for studies to investigate in vivo degradation as well as potential acid hydrolysis of FOS and GOS through the stomach and small intestine of healthy subjects with analysis of their final DP to verify their intact arrival in the colon. Intestinal catheters proved to be valuable tools to study digestion in the human intestine.^40^

We have recently published two feasibility trials,^41^ in which we focused mainly on FOS and GOS fermentation, including fermentation metabolites and the microbiota composition, inside the human intestine, and host response. Intestinal samples were collected over time after consumption of a drink with FOS and GOS. In this publication, we extend these observations by detailed reporting of the degradation of FOS and GOS using in-depth chemical analyses, including the breakdown kinetics of the digestible mono- and dimer fractions in these mixtures in the distal small intestine or proximal colon of healthy men. We provide direct evidence in humans on the resistances to degradation of prebiotic compounds with specific linkages, monomer compositions, and sizes, opening the future development of new tailored (potential) prebiotics.

## 2 Materials and Methods

Data were collected in two previously performed human clinical trials, of which the methodology was described in detail elsewhere.^41^ Both studies were approved by the Medical Ethics Committee of Wageningen University and registered at ClinicalTrials.gov, identifiers: NCT04013607 (study 1) and NCT04499183 (study 2). All subjects gave written informed consent. The data of FOS and GOS degradation in both studies are jointly analyzed and presented in the current study.

### 2.1 Study Subjects

Male Dutch subjects with an age between 18 and 60 years and a BMI between 18.5 and 30 kg/m^2^ were included. The main exclusion criteria were having a history of medical or surgical events, the use of any prescribed or nonprescribed medication during the 3 weeks prior to study start, smoking, use of pro- pre-, or antibiotics within 3 months before the study started, having less than three bowel movements per week, and excessive alcoholic consumption (i.e., >21 servings per week^42^). They were not lactose intolerant. All subjects filled out a food frequency questionnaire (FFQ) to determine their habitual dietary intake. Six subjects with measurements in the distal ileum are referred to as distal ileum1–6, and one subject with measurements in the proximal colon is referred to as proximal colon1.

### 2.2 Study Design

All details about the study designs and study logistics have been described previously.^41^ In short, study 1 was an acute feeding test day, and before the test day, participants followed a habitual diet. Study 2 was a 7-day parallel intervention with either 15 g/d NDCs or isocaloric maltodextrin, followed by the same acute feeding test day as that in study 1. The 7-day intervention study was found not to affect the luminal microbiota and was therefore not further researched in this publication. One day before the acute feeding test day, subjects were intubated with a 300-cm long nasointestinal catheter with a 1.9 mm aspiration channel (Mui Scientific, Ontario, Canada) that progressed toward the distal small intestine or proximal colon using an inflatable balloon. The next morning, after an overnight fast, subjects visited the hospital again for test day. Subjects consumed a liquid bolus with the NDCs. Afterward, subjects were not allowed to eat or drink, except water. 120 min after NDC bolus consumption, an intraintestinal infusion was delivered with a total volume of 20 mL, which was described previously.^41^ Using the catheter aspiration channel, we aimed to collect luminal samples at baseline, 60, 90, 120, every 20 min between 130 and 310 min (study 1) or between 130 and 390 min (study 2), and every 40 min between 310 and 490 min (study 1 only). Intestinal luminal content was collected using 5 cc syringes in 5 mL tubes, thoroughly mixed, and divided into aliquots which were put on dry ice immediately and stored at −80 °C.

### 2.3 NDC Bolus

**Figure 1:**
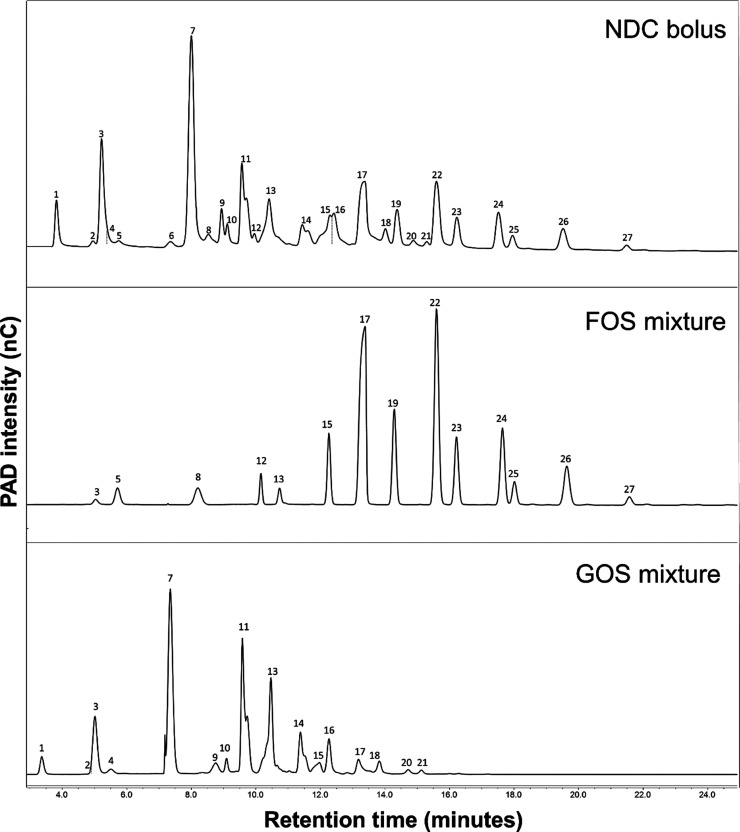
HPAEC-PAD elution patterns of the NDC bolus, the FOS mixture, and the GOS mixture. The peaks are numbered 1–27, the corresponding compounds are described in the Supporting Information Table 1. FOS, fructo-oligosaccharides; GOS, galacto-oligosaccharides; NDC, nondigestible carbohydrates; PAD, pulsed amperometric detection.

The NDC bolus (Figure 1) consisted of 5.4 g of chicory FOS (Frutalose OFP; Sensus, The Netherlands) and 7.1 g of GOS (Vivinal DOMO GOS, FrieslandCampina, The Netherlands: 30% mono- and dimers) to reach a 1:1 ratio of FOS and GOS oligosaccharides (5 g each) in the final bolus in 200 mL of tap water. Additionally, 5 g of nondigestible marker polyethylene glycol 4000 (PEG-4000, Dulcosoft, Sanofi-Aventis, Germany) was dissolved in the bolus. Frutalose OFP contains 93% oligosaccharides with a DP ≤ 10, and 7% fructose, glucose, and sucrose. Vivinal GOS contains 70% oligosaccharides with a DP ≤ 6 and 30% glucose, galactose, and lactose, of which there is around 20% lactose. In total, the NDC bolus contained a mean amount (±SD) of 0.36 ± 0.00 g of glucose + galactose, 0.26 ± 0.22 g of fructose, 1.7 ± 0.46 g of lactose, and 0.41 ± 0.09 g of sucrose. The water-soluble PEG-4000 is not absorbed or metabolized in the GI-tract^43^ and was therefore used to correct for removal of FOS and GOS from the sampling location by transit time rather than degradation.

### 2.4 Measurement of the Carbohydrates in Intestinal Contents

Luminal samples were analyzed for their mono-, di-, and oligosaccharide profiles by ICS3000 high performance anion exchange chromatography with pulsed amperometric detection (Dionex Corp., Sunnyvale, CA, USA). The HPAEC-PAD system, columns, and elution conditions were used as described elsewhere.^44^ In short, the separation was performed using a 2 × 50 mm CarboPac PA-1 guard column followed by a 2 × 250 mm CarboPac PA-1 column using a flow rate of 0.3 mL/min. The elution gradient begin with a linear gradient of 0.02–0.05 M NaOH in 3 min, and 0.05–0.075 M NaOH in 10 min, succeeded by isocratic elution with 0.1 M NaOH for 2 min, and a 50 min gradient of 0–1 M NaOAc in 0.1 M NaOH. Hundred microliters luminal content was centrifuged (10 min, 4 °C, 15,000*g*). The supernatants of most of the samples from subjects distal ileum1 were 10× diluted, distal ileum2 50× diluted, distal ileum3 10× diluted, distal ileum4 300× diluted, distal ileum5 200× diluted, distal ileum6 200× diluted, and from the proximal colon1 100× diluted. The dilution factor was based on a premeasurement. A range of dilutions of the NDC bolus (50–200 μg/mL) was included in the run to cover the linear range of each compound in the bolus. Identification of individual FOS and GOS isomers was partly based on commercial standards. For identification of FOS and GOS isomers for which commercial standards were not available, the elution profiles of the luminal content were compared with the elution profiles of Frutalose FOS (50–200 μg/mL), Vivinal GOS (50–200 μg/mL), Vivinal GOS DP fractions (DP2, DP3, DP4, and DP5), and FOS and GOS profiles characterized in previous research.^18,21^ The standards of the constituent DPs of GOS were obtained previously by size-exclusion chromatographic fractionation of Vivinal GOS.^17^ We relied on the tentative identification of GOS (DP2) compounds described in previous studies.^18,45^ Quantification of glucose, galactose, fructose, sucrose, lactose, 1-kestose, 4-galactosyllactose, and 6-galactosyllactose was possible by including these as standards (Sigma-Aldrich) in the range of 4–20 μg/mL. The data were analyzed with Chromeleon 7.2 SR4 software. The area of each peak was quantified, and the peak areas were normalized to the total NDC area of that specific sample to calculate the relative abundance. The total peak area of compounds from FOS and GOS mixtures that coeluted in one peak were included in both the analysis of FOS and GOS. The percentage recovery of the NDC compounds in the intestine compared to the NDC bolus was estimated using the following formula: [(NDC compound in the intestine/PEG in the intestine)/(NDC compound in the bolus/PEG in the bolus)]*100%.

### 2.5 Measurement of the Nonabsorbable Marker in Intestinal Contents

Concentrations of PEG-4000 were quantified using an anti-PEG sandwich ELISA assay. In short, plates (Nunc MaxiSorp) were coated with 50 μL per well with 5 μg/mL of rat5M-PABM-A anti-PEG antibody (IBMS Academia Sinica, Taiwan) in coating buffer (5.3 g/L Na_2_CO_3_, 4.2 g/L NaHCO_3_, pH 8.0) overnight (4 °C, shaking at 50 rpm). Plates were washed five times with 1× phosphate-buffered saline (PBS) and blocked with 200 μL 1% BSA/1× PBS per well for 2 h at room temperature. Tween was not added to the washing buffer, because its structure is similar to PEG-4000, and therefore interferes with the assay. PEG-4000 standards (0.1–10,000 μg/mL) and samples were diluted in buffer (1% BSA/1× PBS). After another washing step, 50 μL standards or 50 μL intestinal content (500 or 1000 times diluted) was added for 1 h at room temperature, while shaking at 50 rpm. To assess matrix effects, known PEG-4000 concentrations were spiked in small intestinal content without PEG that was diluted 10, 100, or 1000× in dilution buffer. Afterward the plates were washed, and 50 μL per well 6.3-PABG-B biotin anti-PEG detection antibody (IBMS Academia Sinica, Taiwan) was added in a concentration of 5 μg/mL in dilution buffer for 1 h at room temperature. After plate washing, 50 μL per well of 0.5 μg/mL streptavidin conjugated to horseradish peroxidase (Jackson Immunoresearch Europe Ltd., UK) in dilution buffer was added for 45 min at room temperature. The plate was washed again, and 100 μL of freshly prepared 0.5 mg/mL **AzBTS-(NH**_**4**_**)**_**2**_ (Sigma-Aldrich) in 100 mM phosphate-citrate buffer was added per well. 0.2 μL/mL of 30% H_2_O_2_ was added to the **AzBTS-(NH**_**4**_**)**_**2**_ substrate solution directly before use. After 8 min of incubation in the dark, absorbance was read at 414 nm.

### 2.6 Presence of Predicted Microbial Genes Related to FOS and GOS Breakdown

Microbiota composition in the luminal content was determined via sequencing of the variable V4 region of the 16S rRNA gene using Illumina HiSeq2500, as described previously.^41^ The predicted functionality of bacteria in the intestinal lumen was compared with the predicted functionality of fecal bacteria. A fecal sample was collected the day before the test day. The abundances of microbial genes were predicted based on the 16S rRNA gene sequences using the phylogenetic investigation of communities by reconstruction of unobserved states algorithm (version PICRUSt2) with default settings, but the minimum alignment was set to 60%.^46^ The mean (±SD) nearest sequenced taxon index, which is the average branch length that separates each amplicon sequence variant (ASV) from a reference bacterial genome, weighted by the abundance of that ASV in the sample, was 0.17 ± 0.16. Within the sample, the abundance of the selected microbial gene was divided by the abundance of the total microbial genes to calculate the relative abundance. Relative abundance in the ileal samples was compared to the relative abundances in feces using the nonparametric Kruskal–Wallis test.

## 3 Results

### 3.1 Subject Characteristics

Data from seven healthy male subjects were used in the analyses, of which the baseline characteristics are summarized in Table 1. The mean age was 34.6 ± 17.4 years (range 19–59). In six subjects, the catheter was located in the distal ileum, at a mean estimated distance of 21 ± 16 cm (range 10–50 cm) from the ileo-cecal valve. In one subject, the catheter was located in the proximal colon. Due to sampling difficulties, particularly in the proximal colon, not at every time point an intestinal sample was collected.

**Table 1:** Baseline Characteristics and Habitual Daily Intake of (macro)nutrients in Healthy Male Subjects^a^

	*n* = 7 subjects
age, years	34.6 ± 17.4
BMI, kg/m^2^	23.8 ± 2.5
total kcal/d	2528.3 ± 207.3
total carbohydrates^b^, g/d	256.8 ± 39.6
mono- and disaccharides, g/d	88.5 ± 36.5
polysaccharides^c^, g/d	168.2 ± 26.3
fiber^d^, g/d	28.8 ± 8.5

a Values are presented as means ± SD, *n* = 7 subjects.

b Dietary fiber is not included in the total carbohydrates.

c Polysaccharides include digestible carbohydrates and low molecular weight fibers.

d Fibers include high molecular weight fibers, insoluble fibers in water, fibers soluble in water and precipitated by 78% ethanol, not low molecular weight fibers (e.g., fructan, GOS).

### 3.2 Characterization of the NDC Bolus

The HPAEC-PAD chromatograms of the NDC bolus and the original FOS and GOS supplements are visualized separately (Figure 1). Some peaks represent compounds coming from both the FOS supplement and the GOS supplement, so-called coelution, namely glucose + galactose (peak #3), GOS DP3+FOS DP2 (peak #13), GOS DP4+FOS DP4 (peak #15), and GOS DP4+DP5+FOS DP3 (peak #17). Most compounds were distinguished to come from either the GOS mixture or from the chicory-derived FOS. The monosaccharides glucose, galactose, and fructose (peaks 3 and 5) constituted approximately 7% of the total NDC bolus compounds, while lactose and sucrose (peaks 7 and 8) accounted for about 20%. GOS DP2 (peaks 1, 4, and 11) represented an average of 14.7% of the NDC compounds, and GOS DP3-DP6 (peaks 2, 6, 9, 10, 14, 16, 18, 20, and 21) made up 14.9%. FOS DP3 (peak 12) was 1.2%, FOS DP4 (peak 22) was 6.3%, FOS DP5 (peaks 19 and 24) was 6.3%, FOS DP6 (peak 23) was 2.2%, and FOS DP6-DP8 (peaks 25, 26, and 27) accounted for 3.64%. The remaining 24.1% were coeluted compounds derived from GOS (DP3-DP5) and FOS (DP2-DP4). An overview of all characterized compounds, including peak identifications with their chemical structures and relative abundances, is presented in the Supporting Information, Table 1. Except for the monomers lactose and sucrose, the quantification was limited to the relative abundance of each component in the total NDC bolus due to the lack of commercial standards.

### 3.3 Fate of FOS and GOS in the Intestine over Time

**Figure 2:**
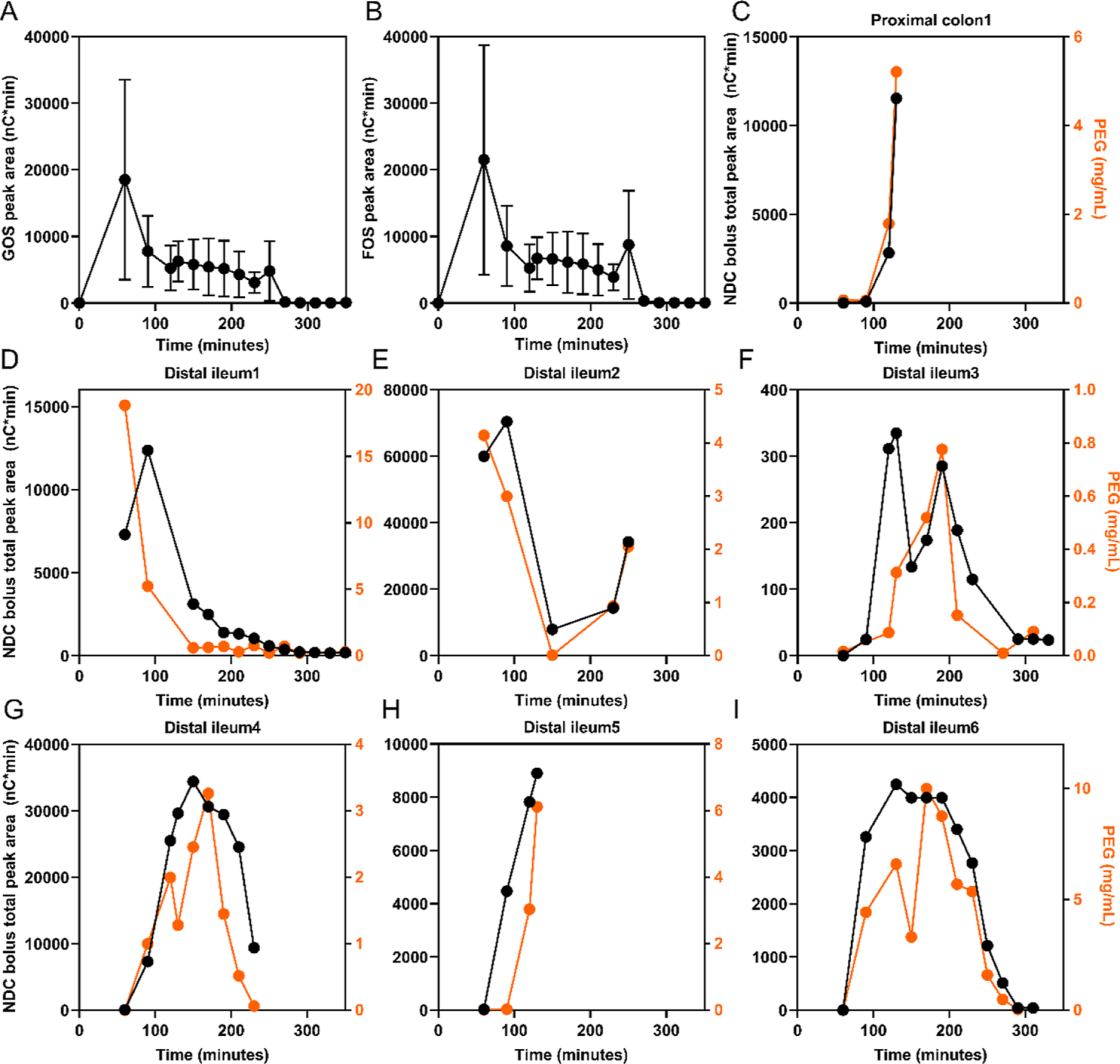
The fate of the NDC bolus compounds in distal ileum or proximal colon of healthy male subjects. The amount of GOS mixture (A), or the FOS mixture (B) is shown as mean ± SD, n = 7 subjects*.* Compounds from FOS and GOS mixtures that coeluted (peak #13, peak #15, and peak #17) are included in both the FOS and GOS peak area. The total peak area of all NDC bolus compounds, and the concentrations of PEG-4000 over time in every subject (C–I). The NDC bolus peak area is shown by the black line (left *y*-axis), and the PEG-4000 concentrations are shown by the orange line (right *y*-axis). The starting time point of appearance differs per individual, depending on when the first sample could be obtained. The digestible carbohydrates of the mixtures, glucose + galactose, fructose, sucrose, and lactose, are excluded from this figure. FOS, fructo-oligosaccharides; GOS, galacto-oligosaccharides; NDC, nondigestible carbohydrates; PEG, polyethylene glycol.

FOS and GOS appeared in the distal ileum or proximal colon within 60–120 min after consumption (Figure 2). The appearance and disappearance of the nondigestible fraction of FOS and GOS were similar (Figure 2A, B), namely, decreasing from 60 to 270 min after bolus consumption, with traces remaining from 270 to 350 min. Within person (Figure 2C–I), the concentration of the NDC bolus compounds over time generally had the same pattern as the concentrations of PEG-4000 over time in the intestine. Overall, the similar behavior of FOS and GOS compared to PEG-4000 implies the removal of FOS and GOS from the aspiration site due to their transit through the small intestine.

#### 3.3.1 FOS in the Intestine over Time

**Figure 3:**
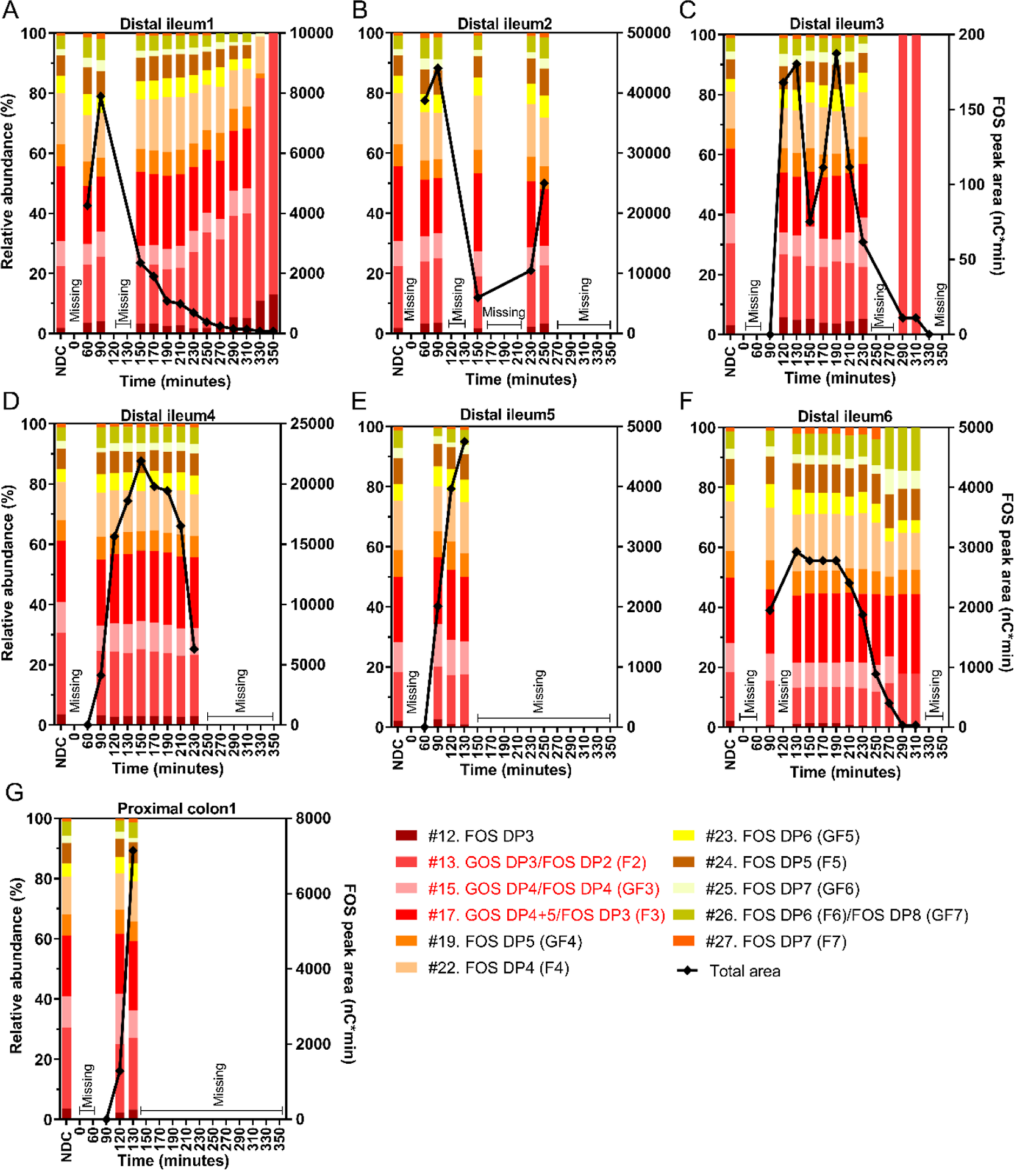
Profile of the compounds originating from the chicory-derived FOS mixture in the distal ileum or proximal colon of healthy male subjects over time after NDC consumption. The relative abundances are shown on the left *y*-axis, and the diamond shapes connected by the black line show the area of compounds from the FOS mixture (right *y*-axis). Compounds GOS DP3/FOS DP2 (F2, peak #13), GOS DP4/FOS DP4 (GF3, peak #15), and GOS DP4 + 5/FOS DP3 (F3, peak #17) coeluted with a compound from the GOS mixture, indicated in red in the legends. The digestible carbohydrates, glucose + galactose, fructose, and sucrose, are excluded from this figure. The numbers in the legends correspond with peaks in the chromatograms in Figure 1. Missing samples were the result of sampling difficulties. DP, degree of polymerization; F, fructose series attached to a fructose moiety; FOS, fructo-oligosaccharides; GF, fructose series attached to a glucose moiety; GOS; galacto-oligosaccharides.

The degradation of individual compounds in the prebiotic fraction of the FOS mixture in the small intestine was evaluated (Figure 3). The mean estimated recovery of this FOS fraction was 96 ± 25% for the *n* = 7 subjects (all time points, range between subjects 78.1 ± 14.4 to 115.1 ± 32.2). Over time, the relative abundances of the FOS compounds in the intestine indeed remained constant and were the same as those in the NDC bolus. Only after 270 min, relatively decreased higher DP fractions and increased GOS DP3+FOS DP2 (F2) (peak #13) was found in three subjects (Figure 3A, F, H). However, the total absolute amounts (black lines in Figure 3) as well as the absolute amounts of specifically peak #13 decreased (Supporting Information, Figure 2) in a similar manner as PEG-4000. This makes it unlikely that GOS DP3 + FOS DP2 was formed upon degradation of compounds with DP ≥ 3. After hydrolysis of constituents in FOS, mainly fructose and to a lower extent glucose would remain, but traces of fructose and sucrose were detected only at the first time points of sampling, in a maximum of two or four subjects, respectively (Supporting Information, Table 2). This indicates no hydrolysis of FOS or most likely fast fructose absorption in the small intestine. Minor shifts in the abundances of FOS compounds DP ≥ 2 were found over time in the distal ileum compared to those ingested, which indicates FOS was mostly resistant to digestion in the small intestine.

#### 3.3.2 GOS in the Intestine over Time

**Figure 4:**
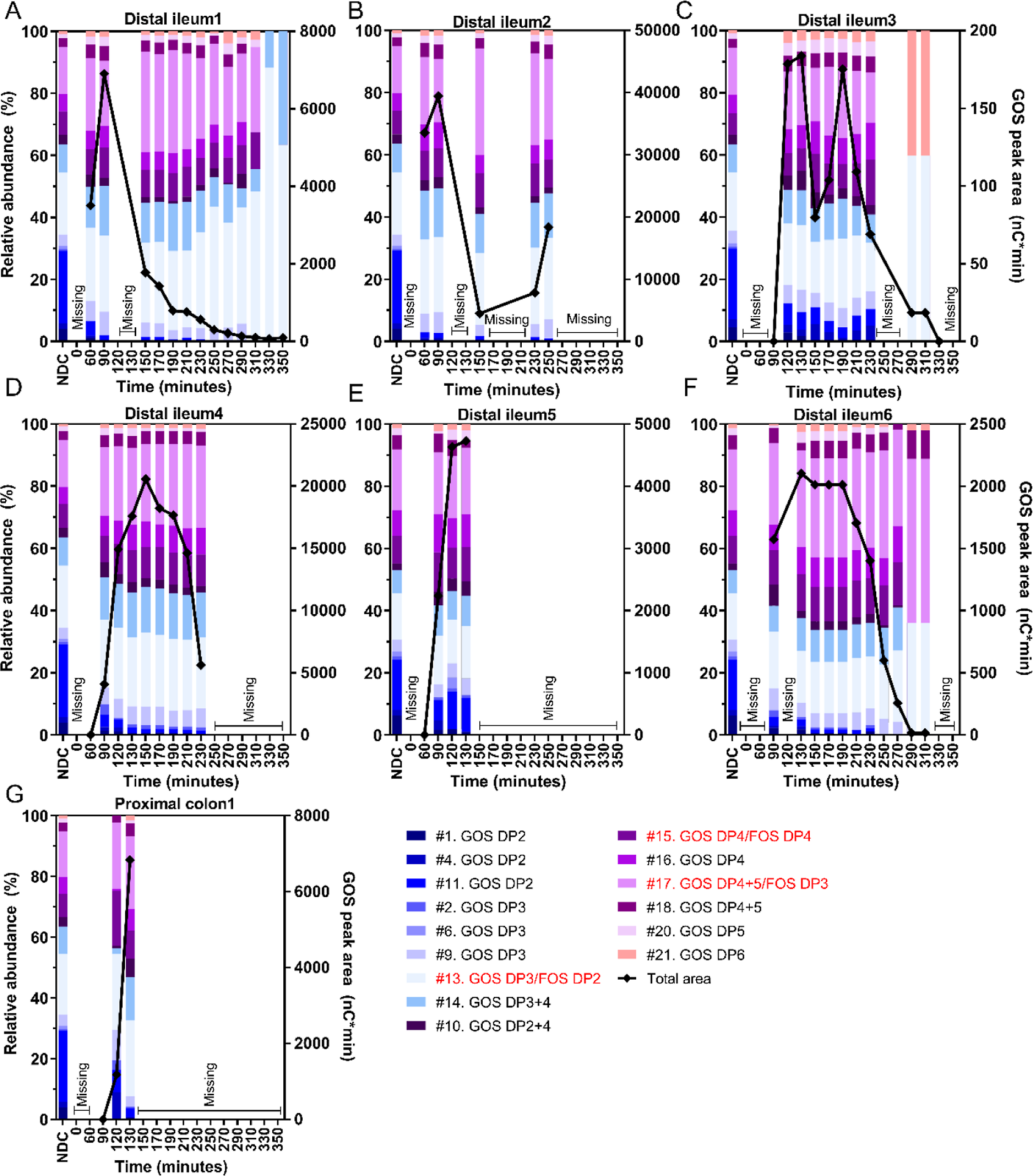
Profile of the compounds originating from the GOS mixture in the distal ileum or proximal colon of healthy male subjects over time after NDC consumption. The relative abundances are shown on the left *y*-axis, and the diamond shapes connected by the black line show the area of compounds from the GOS mixture (right *y*-axis). Compounds GOS DP3/FOS DP2 (peak #13), GOS DP4/FOS DP4 (peak #15), and GOS DP4 + 5/FOS DP3 (peak #17) coeluted with a compound from the FOS mixture, indicated in red in the legends. The digestible carbohydrates, namely glucose + galactose and lactose, are excluded from this figure. The numbers in the legends correspond with peaks in the chromatograms in Figure 1. Missing samples were the result of sampling difficulties. DP, degree of polymerization; F, fructose series attached to a fructose moiety; FOS, fructo-oligosaccharides; GF, fructose series attached to a glucose moiety; GOS, galacto-oligosaccharides.

We evaluated the degradation of individual compounds in the GOS mixture in the small intestine (Figure 4). The digestible carbohydrates in this mixture, glucose, galactose, and lactose, are excluded from this figure to visualize changes in the prebiotic fraction (GOS DP2–6). The mean estimated recovery of GOS was 76 ± 28% for the *n* = 7 subjects (all time points, range between subjects 65.0 ± 28.2% and 136.6 ± 79.6%), which indicates that some degradation occurred in the small intestine. When comparing the relative abundance profiles in the intestine to those in the bolus, it is clear that GOS DP3–6 remained unchanged before 250 min. In contrast, the relative abundance of the prebiotic GOS dimers (DP2 fraction, peaks #1, #4, #10, and #11) decreased in the small intestine of all subjects. After 250 min, in three participants (Figure 4, A, C, F), the relative abundances changed, specifically characterized by a relative increase in peak #13. However, both the total absolute amounts and peak #13 decreased (Figure 4, black lines; Supporting Information, Figure 2), making it more likely that this shift was caused by differences due to flow behavior and detection limits. Traces of glucose + galactose, as well as lactose, were detected in the distal ileum or proximal colon of all subjects over time (Supporting Information, Table 2). In the ileum of four subjects, negligible concentrations of glucose + galactose were measured already before the arrival of other NDC bolus constituents. Overall, a lowered abundance of the prebiotic GOS DP2 fraction was found in the distal ileum and proximal colon, while the abundances of GOS DP3–6 did not change.

#### 3.3.3 GOS DP2 Compounds in the Intestine over Time

**Figure 5:**
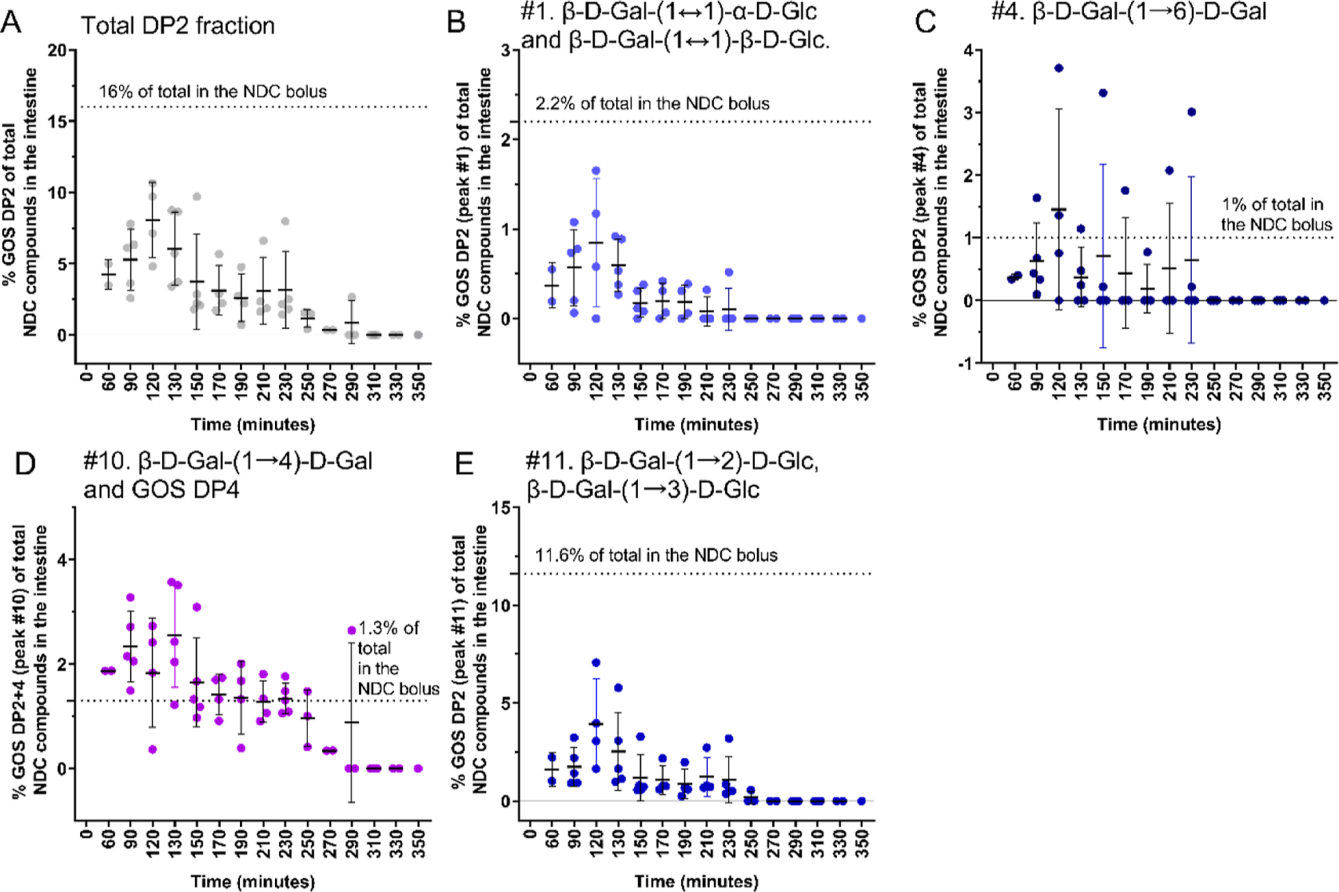
Relative abundances of the GOS dimers in the distal ileum or proximal colon of healthy male subjects over time. (A) The total GOS DP2 fraction, (B) GOS DP2 peak #1, (C) GOS DP2 peak #4, (D) GOS DP2 + 4 (peak#10), and (E) GOS DP2 (peak #11) as a percentage of all NDC compounds detected in the intestine over time. The means ± SDs are shown, *n* = 7 subjects. The dots show the individual values. The dotted line indicates the GOS DP2 mean relative abundance (%) in the NDC bolus (*n* = NDC boluses). Lactose is excluded from the DP2 fraction. DP, degree of polymerization; Gal, galactose; Glc, glucose; GOS, galacto-oligosaccharides.

Since especially the GOS DP2 fraction decreased during transit in the small intestine, we have plotted the kinetics of all GOS dimers separately (Figure 5, *n* = 7 subjects). The mean relative abundance of the total GOS DP2 fraction in the distal ileum after NDC consumption was lower compared with those in the NDC bolus (Figure 5A). Especially the relative abundances of β-D-gal-(1↔1)-α-D-glc+β-D-gal-(1↔1)-β-D-glc (Figure 5B) and β-D-gal-(1 → 2)-D-glc+β-D-gal-(1 → 3)-D-glc (Figure 5E) were decreased. Also, the absolute amounts of these dimers in the intestine were reduced (Supporting Information, Figure 1). The mean estimated recoveries (i.e., arrival) in the distal ileum or proximal colon at time points 60–130 min after consumption were 22.8 ± 11.1% for β-D-gal-(1↔1)-α-D-glc+β-D-gal-(1↔1)-β-D-glc and 19.3 ± 19.1% for β-D-gal-(1 → 2)-D-glc+β-D-gal-(1 → 3)-D-glc (Supporting Information, Table 3). In contrast, β-D-gal-(1 → 6)-D-gal (Figure 5C) and β-D-gal-(1 → 4)-D-gal + GOS DP4 (Figure 5D) had higher recoveries, namely, 43.7 ± 24.6% and 68.0 ± 38.5%, respectively (Supporting Information, Table 3). Overall, the degradation of the GOS DP2 fraction was dependent on the type of linkage between the monomers, with β(1 → 6) and β(1 → 4) linked dimers being more resistant to degradation in the small intestine than β(1↔1) and β(1 → 2)+β(1 → 3) linked dimers.

#### 3.3.4 Lactose in the Intestine over Time

**Figure 6:**
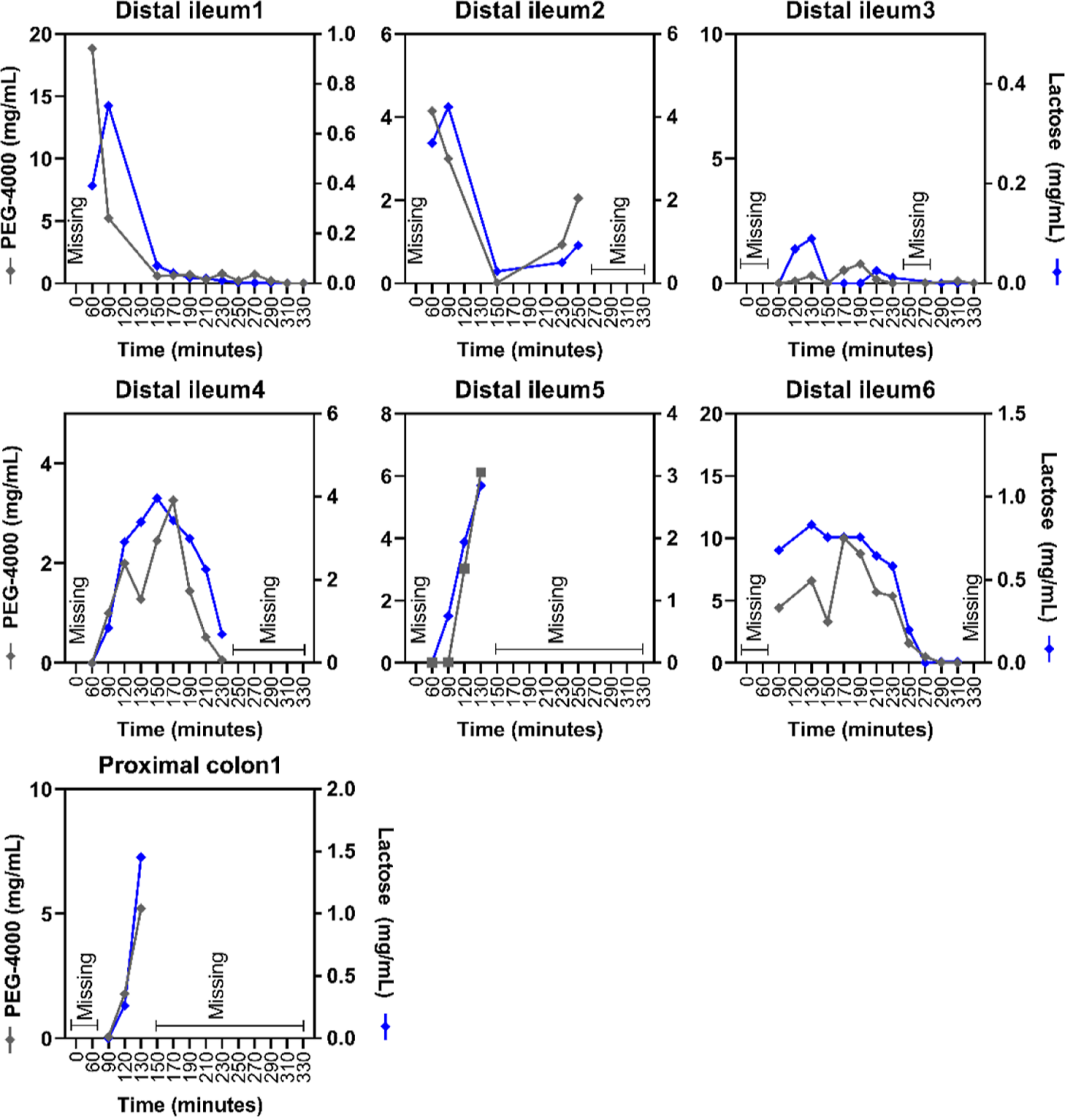
Presence of lactose over time in the distal ileum or proximal colon of healthy male subjects over time. The PEG-4000 concentration is shown by the gray line on the left *y*-axis, and the lactose concentration is shown by the blue line (right *y*-axis). Lactose originated from the GOS mixture. Missing = no intestinal sample could be collected at this time point. PEG, polyethylene glycol.

Lactose can be digested in the small intestine by brush-border enzyme lactase. Figure 6 illustrates the lactose concentrations in the distal ileum and proximal colon of all subjects over time. The initial mean estimated lactose recovery in the intestine was 42.1 ± 0.3% at 60 min, 40.1 ± 4.5% at 90 min, 40.0 ± 7.0% at 120 min, and 36.3 ± 7.9% at 130 min (Supporting Information, Table 3). Furthermore, the decrease in lactose over time (Figure 6, blue line) followed the decrease of PEG-4000 (Figure 6, gray line). This shows the removal of lactose from the aspiration site by peristalsis and not digestion. The NDC bolus contained a mean amount (±SD) of 1.7 ± 0.46 g lactose (8.5 mg/mL). Even though we included lactose tolerant subjects, a fraction of lactose likely coming from the 1.7 g lactose in the NDC bolus, was recovered at the end of the small intestine or in the proximal colon.

### 3.4 Presence of Predicted FOS- or GOS-Degrading Enzymes in the Intestinal Samples

Finally, we aimed to address the potential role of small intestinal microbiota in the hydrolysis of the GOS dimers. Hence, selected microbial genes were derived from the total predicted genome, which was predicted based on the microbiota composition (16S rRNA gene sequencing data). We compared the relative abundance of microbial β-galactosidase in the ileal samples and feces (Table 2). Feces are used as comparison because it has been shown that human fecal bacteria efficiently break down FOS and GOS in vitro, and hence, we expected a higher predicted abundance. The predicted β-galactosidase relative abundance was significantly lower in ileum microbiota (0.245 ± 0.109%) compared to fecal microbiota (0.506 ± 0.108%). The relative abundance of microbial gene fructan β-fructosidase, involved in FOS breakdown, was significantly lower in the ileum versus fecal microbiota, while sucrase had a significantly higher relative abundance compared to fecal microbiota.

**Table 2:** Abundance^a^ of Selected Microbial Genes Involved in the Breakdown of FOS and GOS of Luminal Content and Feces of Healthy Male Subjects

microbial gene	EC number	KO number	relative abundance distal ileum,% (*n* = 6 subjects, 52 samples)	relative abundance feces,% (*n* = 7 subjects)	*P*-value^b^
GOS breakdown					
β-galactosidase/β-d-galactohydrolase	EC 3.2.1.23	K01190/K12111/K12308/K12309	0.245 ± 0.109	0.506 ± 0.108	0.000^a^
FOS breakdown					
fructan β-fructosidase/β-d-fructohydrolase^c^	EC 3.2.1.80	K03332	0.016 ± 0.018	0.042 ± 0.025	0.001^a^
sucrase/β-fructofuranosidase^d^	EC 3.2.1.26	K01193	0.236 ± 0.146	0.097 ± 0.030	0.000^a^

a Data are presented as mean relative abundance (%) of the total genes present in the predicted microbial genome of the sample ± SD.

b The abundances of the selected genes relative to the total genes in the ileum and feces were compared using a nonparametric independent samples test. Because of the high variability in microbiota composition during the day, all samples collected in the intestine are treated as an independent observation.

c Hydrolysis of terminal, nonreducing (2 → 1) β-d-fructofuranose residues in fructans and sucrose.

d The substrate includes sucrose. Endoinulinase (EC 3.2.1.7) was not detected in the data set. EC, Enzyme Commission number; FOS, fructo-oligosaccharides; GOS, galacto-oligosaccharides; KO, KEGG Ortholog.

## 4 Discussion

We investigated the degradation of all constituents of FOS and GOS in the human small intestine in detail, including the digestible mono- and dimers. The relative abundances of FOS compounds in the distal ileum or proximal colon of all subjects were comparable to those ingested, whereas a reduction in the number of GOS dimers was observed. The digestible dimer lactose present in the GOS mixture was still partly present at the end of the small intestine or in the proximal colon of most participants.

### 4.1 FOS are not Degraded in the Distal Small Intestine

It has been shown in a previous study that only the GF3 fraction of FOS was slightly subjected to degradation in an in vitro static digestion model (2 h incubation, 4–6% hydrolysis).^47^ In another previous in vitro study, using rat small intestine extract, 15% hydrolysis of FOS after 120 min of digestion was reported.^33^ Based on these findings, we expected minor degradation of FOS in the human small intestine. Indeed, we showed that 96% of FOS was recovered in the distal small intestine or upon arrival in the proximal colon. In healthy and ileostomy subjects slightly lower recoveries from the small intestine were reported for FOS (89 ± 9%,^37^) or inulin (87 ± 4%,^38^), respectively. These recoveries were calculated based on the total ileostomy effluent excretion,^38^ or after infusing known PEG-4000 concentrations at a constant rate proximal to the aspiration site to estimate the total ileal output, and consequently the total output of FOS.^37^ The profiles of both FOS F2–F7 fractions (Fn series) and FOS GF2-GF7 fractions (GFn series) in the human small intestine were comparable to the ratios in the NDC bolus. The stable profiles in this study clearly indicated a negligible breakdown of FOS, which is in line with previous findings for FOS GF2, GF3, and GF4.^37^ When digestive enzymes or microbiota degrade fructans, a specificity toward lower DP compounds can be expected.^31,33,48,49^ In contrast to the GOS dimer degradation, we did not find a breakdown of FOS dimers (F2). This shows the resistance of β-(2,1) linked fructose units toward degradation. This confirms that not only DP and linkages between monomers determine resistance toward degradation but also the monomer composition. We did not detect fermentation end products, the SCFAs, in the same samples collected from the intestine.^41^ Overall, FOS is minimally or neither digested by host enzymes nor hydrolyzed,^32,33,50^ nor absorbed,^37^ nor fermented by bacteria in the human small intestine.

### 4.2 Linkage- and Size-dependent GOS DP2 Digestion in the Human Small Intestine, without Digestion of DP ≥ 3

To the best of our knowledge, we are the first to study the degradation of GOS in the small intestine of human subjects. Several studies using in vitro static carbohydrate digestion models showed that GOS was hydrolyzed by small intestine brush-border enzymes from pigs or rats within 2 h, namely 34%^33^ and 33%^35^ using rat enzymes, or 23–50% (dependent on the type of linkage) using pig enzymes.^36^ Based on these findings and the mean human intestinal transit time, some digestion by the brush-border enzymes was expected.^35^ Indeed, the assumed prebiotic and nondigestible GOS DP2 fraction was degraded in a glycosidic-linkage dependent manner, in line with previous findings in rats.^51^ GOS β(1↔1) and β(1 → 2)+β(1 → 3)-linked dimers showed higher degradation of 77 and 81%, respectively, than GOS β(1 → 4) and β(1 → 6)-linked dimers (32 and 56%, respectively). This linkage-specific breakdown can be clarified by the binding site of carbohydrases that better accommodates certain glycosidic linkages.^52^

As previously shown, the small intestine bacteria can also ferment GOS^31,53^ with 31–82% degraded before 5 h in vitro,^31^ but we did not detect fermentation end-products upon FOS/GOS consumption in the ileum, as published before.^41^ In this study, it was not possible to differentiate between digestion by host lactase, which is a type of galactosidase, or degradation by microbial galactosidases, since lactase may be released into the intestinal lumen.^54−56^ Another explanation for the decreased amounts of GOS dimers could be the passage of intact di- or oligosaccharides across the intestinal wall as shown before,^57−60^ but we did not analyze the appearance of GOS in the blood or urine. In contrast to a study in rats,^51^ we showed that in the human intestine the relative abundances of GOS DP3–6 did not change compared to those ingested via the bolus. This discrepancy may be explained by the small differences in hydrolyzing activity of disaccharidases between animals and humans.^61^ Overall, we show linkage-dependent GOS dimer degradation, while GOS DP ≥ 3 is not degraded in the small intestines of healthy subjects.

### 4.3 Glucose and Galactose Presence in the Distal Small Intestine

Glucose and/or galactose were detected in the distal ileum or proximal colon of all subjects over time. Glucose and galactose could have originated from the consumed NDC bolus, although absorption takes place in the (proximal) jejunum at rates between 0.15 and 0.3 g/min.^62−64^ The NDC bolus contained only 0.36 g of glucose + galactose, which was expected to be absorbed within minutes. Their presence could also have resulted from GOS DP2 breakdown (i.e., consisting of glucose and galactose monomers). A more likely explanation is interference in the analysis due to host compounds, for instance, mucus saccharides, in the intestinal aspirates with the same elution time as glucose + galactose. This hypothesis is corroborated by the finding that in four subjects, low concentrations of glucose + galactose were measured already before the arrival of other NDC constituents. However, four mucus sugars, galactosamine, glucosamine, *N*-acetylglucosamine, and *N*-acetylgalactosamine, did not interfere with glucose + galactose detection. We may have sampled other (unknown) mucus or host digestive compounds while aspirating from the intestinal catheter.

### 4.4 Lactose Presence in the Distal Small Intestine of Healthy Dutch Adults

Surprisingly, some lactose was still recovered at the end of the small intestine or in the proximal colon of all of the participants. Since we did not observe breakdown of GOS DP ≥ 3, the lactose fraction is expected to originate from the NDC bolus. Lactose is degraded by host lactase, highly abundant in the proximal jejunum and gradually declining toward the ileum.^65,66^ Therefore, we did not expect to detect lactose in the distal ileum or proximal colon. There was an initial loss of lactose after passage through the small intestine (52.5–72.1%), while 27.9–47.5% from the 1.7 g of ingested lactose was still present. Afterward, lactose removal is expected due to peristalsis rather than digestion in the distal ileum, because the removal of lactose was constant to the decrease of PEG-4000. The amount of lactose in the NDC bolus was much lower than the dose, 12–18 g, usually reported giving problems in lactose-intolerant persons,^27^ which were excluded in this study. All participants indicated in the FFQ that they consume dairy products, for instance, milk or yogurt, without complaints such as bloating or flatulence. It is not possible to conclude a relation between age and lactose recovery, as only three subjects were above the age of 35. The FOS and GOS supplements, including lactose, were dissolved in only water, which may have resulted in a rapid GI transit. It is known that when ingested via food, the intestinal content will have different physical characteristics, flow behavior (mixing), and transit time,^67^ with consequent effects on nutrient digestion. Our test conditions may have limited the diffusion of lactose from the lumen to the mucosal epithelium.^68^ There is no literature stating that intestinal catheters cause nutrient malabsorption or influence digestive processes, although intubations may have decreased the small intestine residence of foods^69^ or changed intestinal motor patterns.^70^ Overall, a portion of the ingested lactose, present in GOS, was detected at the end of the ileum or in the proximal colon of healthy Dutch subjects.

### 4.5 Study Strengths and Limitations

Our study has several limitations that impact the interpretation of the results. There was significant interindividual variability and a small sample size, largely due to high drop-out rates and inconsistent sampling times, with participants completing varying numbers of sampling points. Furthermore, the study involved mostly young participants and exclusively Dutch males, limiting the generalizability of the findings across different sexes, ages, and ethnicities. We provided FOS and GOS together in one drink. By using HPAEC-based characterization, we were able to distinguish most compounds derived from either FOS or GOS, but not all due to coelution. Moreover, due to the coelution of GOS isomers and oligomers with a different DP, not all individual GOS compounds in the complex GOS mixture could be annotated.^71^ Future research could benefit from applying a characterization method based on UHPLC-MS using a porous graphitic carbon column to further zoom in to individual GOS components.^17^ The used nonabsorbable marker in this study, soluble PEG-4000, showed comparable flow behavior as FOS and GOS in the GI-tract, even though the molecular weight of PEG-4000 (∼4000 g/mol) is greater than those of FOS and GOS (e.g., FOS DP5: 828.7 g/mol). In human trials, PEG-4000 is commonly used and quantified in intestinal contents using a turbidimetric method as already proposed by Hyden et al. decades ago.^72^ As the turbidity of intestinal samples differed over time and is expected to be influenced by other factors besides only PEG, we used a more direct measurement to quantify PEG-4000. We detected PEG-4000 using high-performance size-exclusion chromatography, but the presence of FOS and GOS interfered with quantification. In the end, we successfully applied a sandwich ELISA assay using a detection antibody that binds directly to the PEG-4000 backbone with a low detection limit (0.1 μg/mL) and without interference from FOS, GOS, or fecal water without PEG.

In this study, we confirmed that in the human small intestine, FOS/oligofructose chains of DP ≥ 2 from chicory roots are not degraded, absorbed, or fermented by small intestinal bacteria. Similarly, the GOS chains of DP ≥ 3 were not degraded in the small intestine of healthy adults. Nowadays there is increased interest in structure–function relationships of NDCs, since depending on the structure they can exert direct immunostimulatory effects through toll-like receptors or directly in immune cells,^49,73,74^ which are present mainly in the small intestine. Hence, the GOS DP ≥ 3 and FOS ≥ 2 structures can exert direct effects in this GI-tract region. GOS dimers were partially degraded or absorbed in the small intestine in a linkage-specific manner, showing the key role of the glycosidic linkage in GOS dimer digestion. Individual compounds with different linkages and DP have been shown to differ in bioactivity for fermentability in the colon with consequent health impact.^21^ One may speculate that studying the effects of GOS dimers derived from lactose on colonic processes is less relevant since these may not all reach the colon as an available substrate in vivo. GOS mixtures can be structurally distinct, dependent on the source of enzymes used for the production.^18^ We tested GOS produced from lactose; thus, our results may not apply directly to for instance GOS produced from lactulose. We provide direct evidence of the resistances of GOS (DP2) with distinct β-linkages in humans, opening the future development of new tailored (potential) prebiotics that fully resist degradation in the small intestine.

## Supporting Information Available

The peak area of the GOS DP2 fractions in the GOS mixture and the PEG-4000 concentrations in the distal ileum or proximal colon of healthy man over time, the presence of the GOS DP3 + FOS DP2 (F2) fraction over time in the distal ileum or colon of healthy man over time after NDC consumption, compounds present in the NDC bolus, concentrations of glucose + galactose, fructose, sucrose, and lactose in the distal ileum or colon of healthy man over time the recoveries of the GOS DP2 fractions and lactose in the distal ileum or proximal colon of healthy man (PDF)
