# Supplementary material for: Detailed Analysis of Prebiotic Fructo- and Galacto-Oligosaccharides in the Human Small Intestine
Source: J Agric Food Chem. 2024 Sep 16;72(38):21152–65. doi: 10.1021/acs.jafc.4c03881 (PMC11440495; doi:10.1021/acs.jafc.4c03881)
Supplement: Supplementary file 1 — jf4c03881_si_001.pdf [file jf4c03881_si_001.pdf]

Supporting information.

Detailed analysis of prebiotic fructo- and galacto-oligosaccharides in the human small intestine.

Mara P.H. van Trijp[1*], Melany Rios-Morales[2], Madelon J. Logtenberg[3], Shohreh Keshtkar[1], Lydia A. Afman[1], Ben Witteman[1,4], Barbara Bakker[2], Dirk-Jan Reijngoud[2], Henk Schols[3], Guido J.E.J. Hooiveld[1]

[1]Division of Human Nutrition and Health, Wageningen University, 6708 WE Wageningen, the Netherlands

[2]Laboratory of Pediatrics, Center for Liver, Digestive and Metabolic Diseases, University of Groningen, University Medical Center Groningen, 9713 GZ Groningen, the Netherlands

[3]Laboratory of Food Chemistry, Wageningen University, 6708 WG Wageningen, the Netherlands

[4]Department of Gastroenterology and Hepatology, Hospital Gelderse Vallei, 6716 RP Ede, the Netherlands

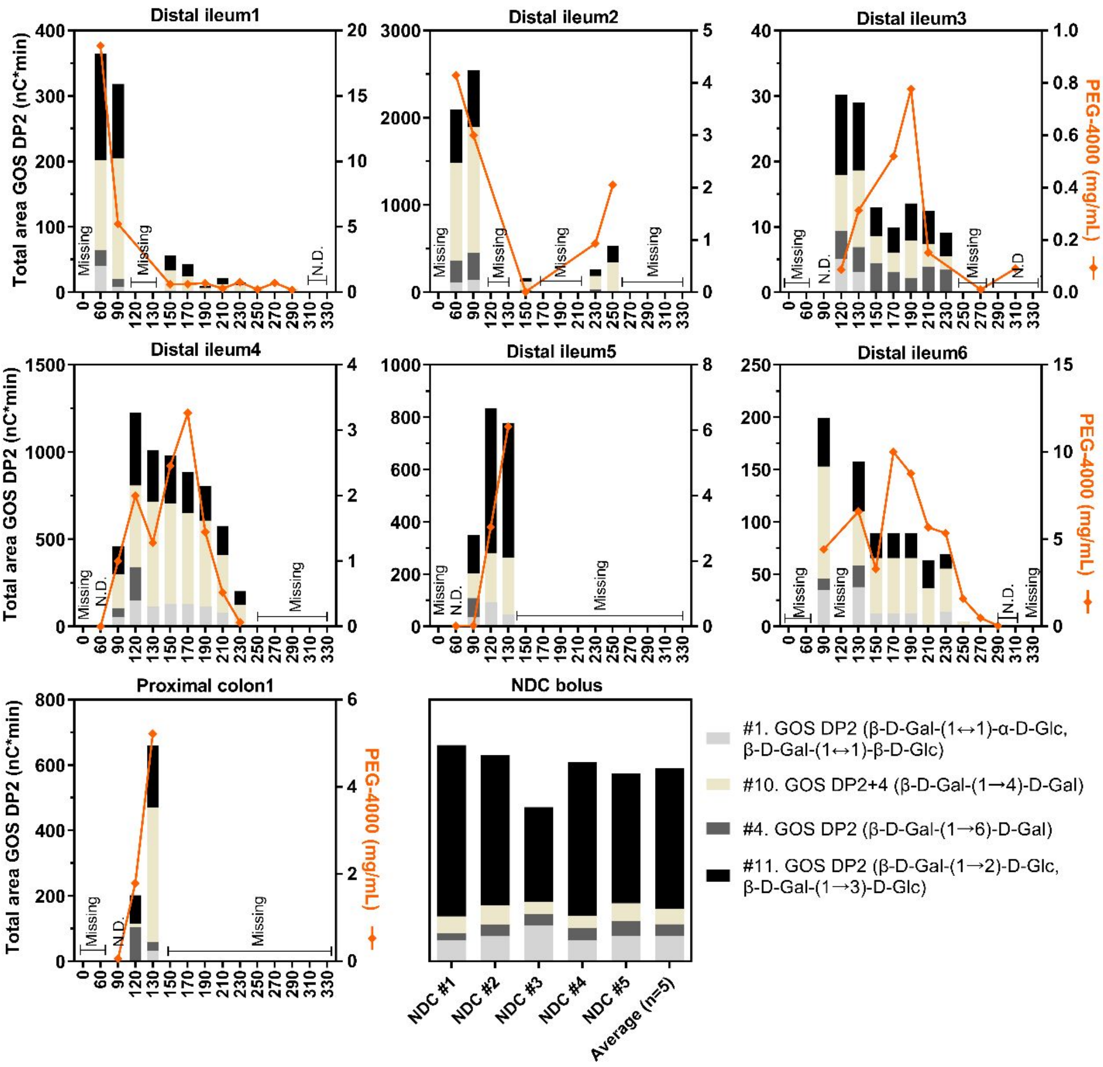

Supplementary Figure 1. The peak area of the GOS DP2 fractions in the GOS mixture and the PEG-4000 concentrations in the distal ileum or proximal colon of healthy man over time. The GOS DP2 peak areas were analyzed by HPAEC-PAD (left y-axis), and the non-absorbable marker PEG-4000 concentrations are shown by the orange line (right y-axis). Missing samples were the result of sampling difficulties. N.D. = GOS DP2 was not detected in this sample. DP, degree of polymerization; GOS, galacto-oligosaccharides; PEG, polyethylene glycol.

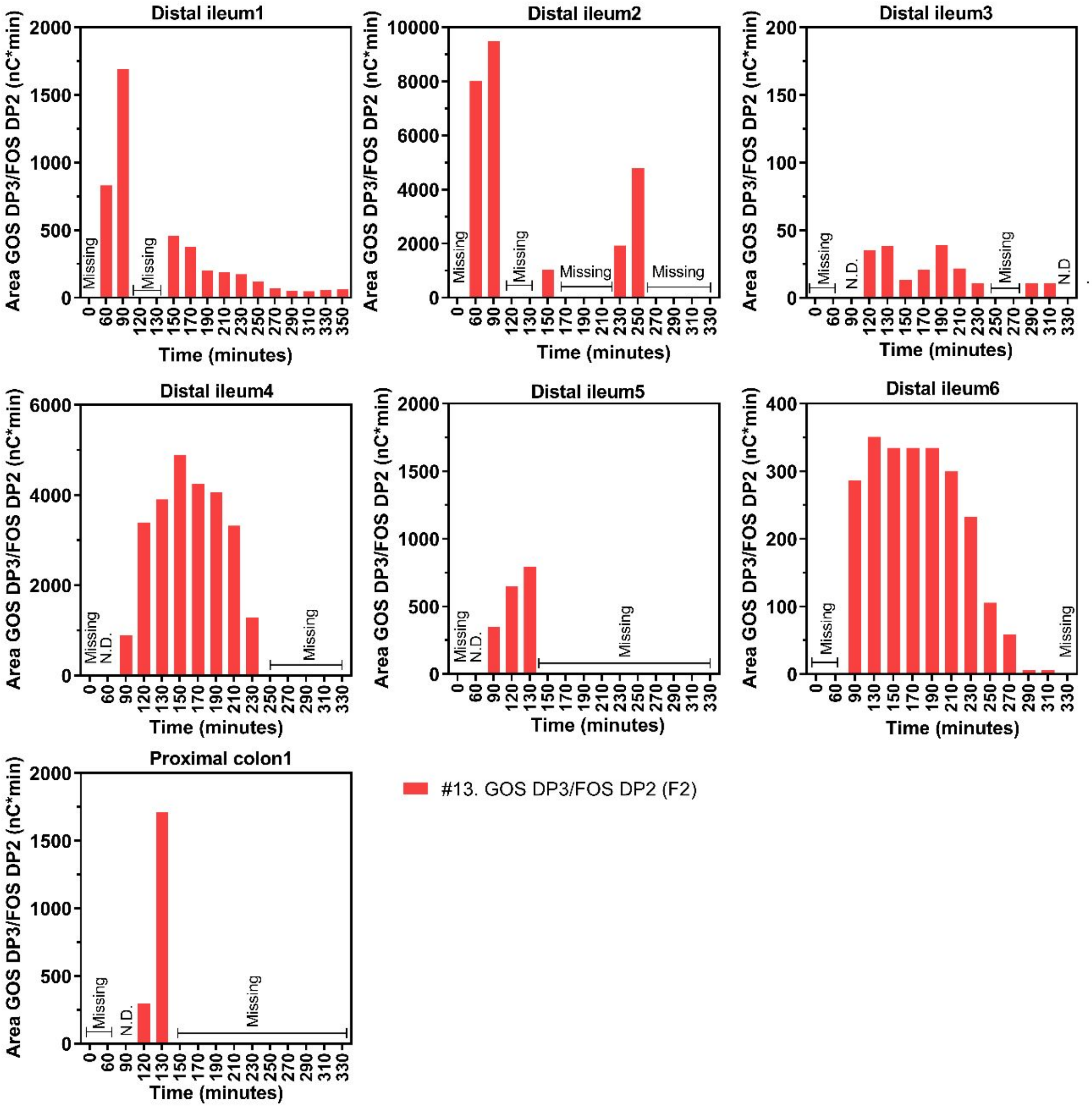

Supplementary Figure 2. The presence of the GOS DP3 + FOS DP2 (F2) fraction over time in the distal ileum or colon of healthy man over time after NDC consumption. This fraction was peak #13 in the chromatogram. N.D. = not detected in this sample. Missing samples were the result of sampling difficulties. DP, degree of polymerization; F, fructose series attached to a fructose moiety; FOS, fructo-oligosaccharides; GOS, galacto-oligosaccharides.

Supplementary Table 1. Compounds present in the NDC bolus[1]. The peak area is expressed as relative abundance compared to the total area of all compounds in the NDC bolus, as analyzed by HPAEC-PAD. The NDC bolus elution pattern is shown in **Error! Reference source not found.**. The sum of all percentages is 100% within sample.

**Peak in chromatogram**	**Identification of peak**	**Structures of FOS compounds**	**Structures of GOS compounds[3]**	**% of total NDC bolus**
1	GOS DP2	-	β-D-Gal-(1↔1)-α-D-Glc<br>β-D-Gal-(1↔1)-β-D-Glc	2.18 ± 0.62
2	GOS DP3	-	No information available	0.30 ± 0.18
3[2]	Glucose + galactose DP1	β-D-Glc*p* (glucose)	β-D-Glc*p* (glucose)<br>β-D-Gal*p* (galactose)	6.39 ± 0.97
4	GOS DP2	-	β-D-Gal*p*-(1→6)-D-Gal*p*	0.98 ± 0.37
5[2]	Fructose DP1	β-D-Fru	-	0.85 ± 0.20
6	GOS DP3	-	No information available	0.51 ± 0.30
7[2]	Lactose DP2	-	β-D-Gal*p*-(1→6)-D-Glc*p* (*allo*-lactose)<br>β-D-Gal*p*-(1→4)-D-Glc*p* (lactose)	17.66 ± 1.40
8[2]	Sucrose DP2	β-D-Fru(2→1)-α-D-Glc	-	1.74 ± 0.35
9	GOS DP3	-	β-D-Gal*p*-(1→4)-[β-D-Gal*p*-(1→6)-]D-Glc*p*<br>β-D-Gal*p*-(1→6)-β-D-Gal*p*-(1→4)-D-Glc*p* (6'galactosyllactose)	1.93 ± 0.38

10	GOS DP2+<br>GOS DP4	-	β-D-Gal*p*-(1→4)-D-Gal*p*<br>No information available about DP4.	1.32 ± 0.39
11	GOS DP2	-	β-D-Gal*p*-(1→2)-D-Glc*p*<br>β-D-Gal*p*-(1→3)-D-Glc*p*	11.56 ± 2.40
12	FOS DP3	GF2: β-D-Fru(2→1)-β-D-Fru(2→1)-α-DGlc<br>(1-kestose)	-	1.23 ± 0.32
13	GOS DP3 +<br>FOS DP2	F2: β-D-Fru(2→1)-β-D-Fru<br>(inulobiose)	β-D-Gal*p*-(1→2)-[β-D-Gal*p*-(1→4)-]D-Glc*p*<br>β-D-Gal*p*-(1→2)-[β-D-Gal*p*-(1→6)-]D-Glc*p*<br>β-D-Gal*p*-(1→3)-[β-D-Gal*p*-(1→6)-]D-Glc*p*<br>β-D-Gal*p*-(1→4)-β-D-Gal*p*-(1→4)-D-Glc*p*<br>(4'galactosyllactose)	10.15 ± 1.81
14	GOS DP3+DP4	-	β-D-Gal*p*-(1→4)-β-D-Gal*p*-(1→2)-D-Glc*p*<br>β-D-Gal*p*-(1→4)-β-D-Gal*p*-(1→3)-D-Glc*p*<br>β-D-Gal*p*-(1→4)-β-D-Gal*p*-(1→2)-[β-D-Gal*p*-(1→4)-]D-Glc*p*<br>β-D-Gal*p*-(1→2)-[β-D-Gal*p*-(1→4)-β-D-Gal*p*-(1→4)-]D-Glc*p*	4.70 ± 0.72
15	GOS DP4 +<br>FOS DP4	GF3: β-D-Fru(2→1)-β-D-Fru(2→1)-β-D-Fru(2→1)-α-DGlc (nystose)	β-D-Gal*p*-(1→4)-β-D-Gal*p*-(1→2)-[β-D-Gal*p*-(1→6)-]D-Glc*p*<br>β-D-Gal*p*-(1→2)-[β-D-Gal*p*-(1→4)-β-D-Gal*p*-(1→6)-]D-Glc*p*<br>β-D-Gal*p*-(1→4)-β-D-Gal*p*-(1→4)-β-D-Gal*p*-(1→6)-D-Glc*p*	4.34 ± 0.41

16	GOS DP4	-	β-D-Gal*p*-(1→4)-β-D-Gal*p*-(1→4)-β-D-Gal*p*-(1→4)-D-Glc*p*	3.12 ± 0.56
17	GOS DP4+DP5 + FOS DP3	F3: β-D-Fru(2→1)-β-D-Fru(2→1)-β-D-Fru (inulotriose)	β-D-Gal*p*-(1→4)-β-D-Gal*p*-(1→4)-β-D-Gal*p*-(1→2)-D-Glc*p*<br>β-D-Gal*p*-(1→4)-β-D-Gal*p*-(1→4)-β-D-Gal*p*-(1→3)-D-Glc*p*<br>β-D-Gal*p*-(1→2)-[β-D-Gal*p*-(1→4)-β-D-Gal*p*-(1→4)-β-D-Gal*p*-(1→6)-]D-Glc*p*<br>β-D-Gal*p*-(1→4)-β-D-Gal*p*-(1→4)-β-D-Gal*p*-(1→2)-[β-D-Gal*p*-(1→6)-]D-Glc*p*<br>β-D-Gal*p*-(1→4)-β-D-Gal*p*-(1→2)-[β-D-Gal*p*-(1→4)-β-D-Gal*p*-(1→6)-]D-Glc*p*<br>β-D-Gal*p*-(1→2)-[β-D-Gal*p*-(1→4)-β-D-Gal*p*-(1→4)-β-D-Gal*p*-(1→6)-]D-Glc*p*<br>β-D-Gal*p*-(1→4)-β-D-Gal*p*-(1→4)-β-D-Gal*p*-(1→2)-[β-D-Gal*p*-(1→6)-]D-Glc*p*<br>β-D-Gal*p*-(1→4)-β-D-Gal*p*-(1→2)-[β-D-Gal*p*-(1→4)-β-D-Gal*p*-(1→6)-]D-Glc*p*	9.64 ± 1.76
18	GOS DP4+DP5	-	β-D-Gal*p*-(1→4)-β-D-Gal*p*-(1→4)-β-D-Gal*p*-(1→4)-β-D-Gal*p*-(1→4)-D-Glc*p*	1.63 ± 0.32
19	FOS DP5	GF4: β-D-Fru(2→1)-β-D-Fru(2→1)-β-D-Fru(2→1)-β-D-Fru(2→1)-α-DGlc (1-β-fructofuranosyl nystose)	-	3.19 ± 0.54

20	GOS DP5	-	β-D-Gal*p*-(1→4)-β-D-Gal*p*-(1→4)-β-D-Gal*p*-(1→4)-β-D-Gal*p*-(1→2)-D-Glc*p*<br>β-D-Gal*p*-(1→4)-β-D-Gal*p*-(1→4)-β-D-Gal*p*-(1→4)-β-D-Gal*p*-(1→3)-D-Glc*p*	0.84 ± 0.23
21	GOS DP6	-	β-D-Gal*p*-(1→4)-β-D-Gal*p*-(1→4)-β-D-Gal*p*-(1→4)-β-D-Gal*p*-(1→4)-β-D-Gal*p*-(1→4)- D-Glc*p*	0.51 ± 0.10
22	FOS DP4	F4: β-D-Fru(2→1)-β-D-Fru(2→1)-β-D-Fru(2→1)-β-D-Fru (inulotetraose)	-	6.32 ± 1.55
23	FOS DP6	GF5: β-D-Fru(2→1)-β-D-Fru(2→1)-β-D-Fru(2→1)-β-D-Fru(2→1)-β-D-Fru(2→1)-α-DGlc	-	2.16 ± 0.50
24	FOS DP5	F5: β-D-Fru(2→1)-β-D-Fru(2→1)-β-D-Fru(2→1)-β-D-Fru(2→1)-β-D-Fru<br>(inulopentaose)	-	3.10 ± 0.46
25	FOS DP7	GF6: β-D-Fru(2→1)-β-D-Fru(2→1)-β-D-Fru(2→1)-β-D-Fru(2→1)-β-D-Fru(2→1)-β-D-Fru(2→1)-α-DGlc	-	1.10 ± 0.18
26	FOS DP6 + FOS DP8	F6: β-D-Fru(2→1)-β-D-Fru(2→1)-β-D-Fru(2→1)-β-D-Fru(2→1)-β-D-Fru(2→1)-β-D-Fru<br>(inulo-hexaose)<br>GF7: β-D-Fru(2→1)-β-D-Fru(2→1)-β-D-Fru(2→1)-β-D-Fru(2→1)-β-D-Fru(2→1)-β-D-Fru(2→1)-β-D-Fru(2→1)-α-DGlc	-	2.08 ± 0.30

27	FOS DP7	F7: β-D-Fru(2→1)-β-D-Fru(2→1)-β-D-Fru(2→1)-β-D-Fru(2→1)-β-D-Fru(2→1)-β-D-Fru(2→1)-β-D-Fru (inulo-eptaose)	-	0.46 ± 0.05

[1]Data is represented as mean ± SD, n=5 NDC boluses used during different test days during two clinical trials. [2]Compound was quantified using commercial standards. [3]Tentative peak identification of a GOS compound based on previous characterization by van Leeuwen et al. (18), Coulier et al. (44), and adopted from Führen et al. (46). DP, degree of polymerization; F, fructose series attached to a fructose moiety; FOS, fructo-oligosaccharides; Fru, fructose; GF, fructose series attached to a glucose moiety; Gal, galactose; Glc, glucose; GOS, galacto-oligosaccharides; NDC, non-digestible carbohydrates.

Supplementary Table 2. Concentrations of glucose+galactose, fructose, sucrose, and lactose in the distal ileum or colon of healthy man over time[1].

Time point (min)	Sample collected in number of subjects	Glucose+galactose (μg/mL[2]), detected in number of subjects (n, %)	Fructose (μg/mL), detected in number of subjects	Sucrose (μg/mL), detected in number of subjects	Lactose (mg/mL), number of subjects
0	n = 0	-	-	-	-
60	n = 4	133 ± 102 (n = 4/4, 100%)	55.1 (n = 1/4, 25%)	15.1 ± 20.8 (n = 2/4, 50%)	0.94 ± 1.6 (n = 3/4, 75%)
90	n = 7	355 ± 408 (n = 7/7, 100%)	60.5 ± 121 (n = 2/7, 29%)	49.5 ± 101 (n = 3/7, 43%)	1.03 ± 1.46 (n = 5/7, 71%)
120	n = 5	422 ± 386 (n = 5/5, 100%)	228 ± 395 (n = 2/5, 40%)	124 ± 160 (n = 4/5, 80%)	1.20 ± 1.20 (n = 5/5, 100%)
130	n = 4	342 ± 243 (n = 4/4, 100%)	138 ± 167 (n = 2/4, 50%)	209 ± 171 (n = 4/4, 100%)	1.94 ± 1.48 (n = 4/4, 100%)
150	n = 5	150 ± 276 (n = 5/5, 100%)	0 (n=0/5, 0%)	103 ± 224 (n = 4/5, 80%)	1.02 ± 1.68 (n = 4/5, 80%)
170	n = 4	173 ± 291 (n = 4/4, 100%)	0 (n=0/4, 0%)	405 (n = 1/4, 25%)	1.06 ± 1.62 (n = 3/4, 75%)
190	n = 4	109 ± 166 (n = 4/4, 100%)	0 (n=0/4, 0%)	88.8 ± 170 (n = 2/4, 50%)	0.94 ± 1.41 (n = 3/4, 75%)
210	n = 4	99.9 ± 148 (n = 4/4, 100%)	0 (n=0/4, 0%)	70.2 ± 135 (n = 2/4, 50%)	0.74 ± 1.05 (n = 4/4, 100%)
230	n = 5	68.3 ± 52.1 (n = 5/5, 100%)	294 (n = 1/5, 20%)	22.0 ± 49.1 (n = 1/5, 20%)	0.36 ± 0.33 (n = 5/5, 100%)
250	n = 3	72.9 ± 70.1 (n = 3/3, 100%)	0 (n=0/3, 0%)	47.6 (n = 1/3, 33%)	0.37 ± 0.48 (n = 3/3, 100%)
270	n = 2	27.1 ± 16.0 (n = 2/2, 100%)	0 (n = 0/2, 0%)	0 (n = 0/2, 0%)	0.0007 ± 0.001 (n = 1/2, 50%)
290	n = 3	7.90 ± 3.77 (n = 3/3, 100%)	0 (n=0/3, 0%)	0 (n=0/3, 0%)	0.003 ± 0.004 (n = 2/3, 67%)

**310**	n = 3	8.92 ± 4.81<br>(n = 3/3, 100%)	0<br>(n=0/3, 0%)	0<br>(n=0/3, 0%)	0.003 ± 0.004<br>(n = 2/3, 67%)
**330**	n = 2	14.1 ± 14.6<br>(n = 2/2, 100%)	0<br>(n = 0/2, 0%)	0<br>(n = 0/2, 0%)	0<br>(n = 0/2, 0%)
**350**	n = 1	23.5<br>(n = 1/1, 100%)	0<br>(n = 0/1, 0%)	0<br>(n = 0/1, 0%)	0<br>(n = 0/1, 0%)
**NDC bolus**		**Glucose+galactose[2]**	**Fructose**	**Sucrose**	**Lactose**
		1816 ± 226<br>µg/mL<br><br>363142 µg/200 mL (total in NDC bolus)	1304 ± 1087<br>µg/mL<br><br>260800 µg /200 mL (total in NDC bolus)	2055 ± 443<br>µg/mL<br><br>441000 µg /200 mL (total in NDC bolus)	8.48 ± 2.05<br>mg/mL<br><br>1695 mg/200 mL (total in NDC bolus)

[1]Data is represented as mean ± SD, n=7 subjects. [2]The concentrations of glucose+galactose were estimated from the glucose standard curve.

Supplementary Table 3. The recoveries of the GOS DP2 fractions and lactose in the distal ileum or proximal colon of healthy man[1].

Subject and age	Time point (min)	Recovery of peak 1 β-D-Gal-(1↔1)-α-D-Glc + β-D-Gal-(1↔1)-β-D-Glc (%)	Recovery of peak 4 β-D-Gal-(1→6)-D-Gal (%)	Recovery of peak 10 β-D-Gal-(1→4)-D-Gal + GOS DP4 (%)	Recovery of peak 11 β-D-Gal-(1→2)-D-Glc + β-D-Gal-(1→3)-D-Glc (%)	Recovery of lactose (%)
Distal ileum1, >35y	60	33.7	64.0	>100	15.3	42.3
	90	3.7	17.6	>100	6.0	43.1
Distal ileum2, <25y	60	11.1	72.0	>100	6.6	41.9
	90	12.0	79.4	>100	6.1	46.1
Distal ileum3, >35y	120	44.9	59.8	99.5	20.4	34.9
	130	25.8	52.1	>100	16.4	43.7
Distal ileum4, <25y	90	26.1	50.6	>100	11.8	36.1
	120	19.3	53.0	>100	11.9	33.3
Distal ileum5, <25y	90	28.3	>100	>100	42.9	39.7
	120	34.9	0	>100	76.5	47.5
Distal ileum6, <25y	90	28.7	26.9	>100	13.7	35.7
	130	19.6	31.7	79.4	9.1	27.9
Proximal colon1, >35y	120	13.6	>100	25.0	25.3	44.3
	130	16.9	17.6	>100	8.6	37.3
Mean	60	22.4±16.0	68.0±5.7	>100	11.0±6.2	42.1±0.3

	90	19.8±11.3	54.9±34.7	>100	16.1±15.4	40.1±4.5
	120	28.2±14.3	37.6±32.7	62.3±52.7	33.5±29.2	40.0±7.0
	130	20.8±4.6	33.8±17.3	89.7±14.6	11.4±4.4	36.3±7.9

[1]The recoveries in the first two samples that could be collected within person during the test day are shown. DP; degree of polymerization, GOS; galacto-oligosaccharide.
